# SARS-CoV-2 coinfections with variant genomic lineages identified by multiplex fragment analysis

**DOI:** 10.3389/fgene.2022.942713

**Published:** 2022-09-26

**Authors:** Richard Lueking, Andrew E. Clark, Madhusudhanan Narasimhan, Lenin Mahimainathan, Alagarraju Muthukumar, Christian P. Larsen, Jeffrey A. SoRelle

**Affiliations:** ^1^ Department of Internal Medicine, Division of Infectious Diseases and Geographic Medicine, University of Texas Southwestern Medical Center, Dallas, TX, United States; ^2^ Department of Pathology, University of Texas Southwestern Medical Center, Dallas, TX, United States

**Keywords:** SARS-CoV-2, co-infection, omicron, fragment analysis, multiplex (RT)-PCR, COVID-19, variant

## Abstract

Immunocompromised patients can experience prolonged SARS-CoV-2 infections in the setting of a lack of protectivity immunity despite vaccination. As circulating SARS-CoV-2 strains become more heterogeneous, concomitant infection with multiple SARS-CoV-2 variants has become an increasing concern. Immunocompromised patient populations represent potential reservoirs for the emergence of novel SARS-CoV-2 variants through mutagenic change or coinfection followed by recombinatory events. Identification of SARS-CoV-2 coinfections is challenging using traditional next generation sequencing pipelines; however, targeted genotyping approaches can facilitate detection. Here we describe five COVID-19 cases caused by coinfection with different SARS-CoV-2 variants (Delta/Omicron BA.1 and Omicron BA.1/BA.2) as identified by multiplex fragment analysis.

## Introduction

Epidemiological surveillance of SARS-CoV-2 has enabled clinical and public health interventions in response to increasing COVID-19 case numbers. These efforts have been aided by broad sequencing/genotyping initiatives, associating case trajectories with the emergence of dominant genetic lineages harboring characteristic mutations. Single nucleotide polymorphisms (SNPs) or insertion/deletion mutations (indels) characterize these divergent strains which exhibit altered cellular tropism, decreased vaccine-derived antibody protection and/or marked increases in transmissibility, morbidity, and mortality. Emergence of these SARS-CoV-2 variants of concern (VOCs) has occurred in succession, with new VOCs often displacing the previous dominant lineage amongst the global population.

RNA viruses accrue mutational changes through multiple mechanisms. Transcriptional errors leading to enhanced mutation rates as a biological consequence of error-prone replication are the most common. Coronaviruses feature the largest viral RNA genomes and utilize an RNA-dependent RNA polymerase (RdRP) devoid of proofreading activity. Due to the rapid speed of the RdRP, mutation rates on the order of 10^−4^–10^−6^ substitutions/nucleotide/round of replication have been reported (Baddock et al., 2022; Moeller et al., 2022). To mitigate these fidelity issues, coronaviruses encode a 3′-5′ exonuclease (ExoN) which confers proofreading ability following genome replication (Moeller et al., 2022). Thus, while SARS-CoV-2 mutates relatively slowly compared to other RNA viruses, including HIV or influenza (Callaway, 2020; Akkiz, 2021), mutagenic change still clearly impacts SARS-CoV-2 epidemiology.

Host immunity (natural or vaccine-derived) also drives the emergence of strains with advantageous mutations (Di Caro et al., 2021). We and others have observed immunocompromised (IC) hosts can exhibit persistently positive SARS-CoV-2 RT-PCR tests long after initial COVID-19 diagnosis, with replication-competent virus recovered in some cases (Sung et al., 2022). Moreover, IC patients often fail to mount robust immunological responses to vaccination (Narasimhan et al., 2021; Thakkar et al., 2021; Vijenthira et al., 2021) and are more susceptible to breakthrough infections (Sun et al., 2022). Additionally, IC hosts may not effectively clear SARS-CoV-2 viral infection leading to accelerated intra-host viral evolution and expanded genomic diversity (Avanzato et al., 2020; Ahmad, 2021; Hensley et al., 2021). Many IC patients also receive immunologic therapies which further increase selective pressure on target SARS-CoV-2 loci (i.e. S-gene), raising the potential for the emergence of more virulent progeny (Chen et al., 2021).

Finally, intra- and interhost recombinatory events enhance coronaviral genomic diversity (Amoutzias et al., 2022; Kozlakidis, 2022). Recombination occurs when host cells are coinfected with different SARS-CoV-2 strains, resulting in the redistribution of genomic material, giving rise to hybrid virions. Identification of this phenomenon in SARS-CoV-2 has proven challenging via traditional bioinformatic pipelines, as lineage evaluation is driven by a limited number of SNPs and coinfections require the circulation of diverse lineages within a population (Kozlakidis, 2022), which are often dominated by a singular VOC. Reinfections with either the same or different variant could also influence VOC emergence, as well as indicate prior SARS-CoV-2 infection does not uniformly provide protective immunity (Babiker et al., 2021). There have been several reports describing SARS-CoV-2 reinfections, but fewer demonstrating reinfection with a genotypically distinct virus (Gupta et al., 2020; Van Elslande et al., 2020; Tillett et al., 2021). Coinfection with two simultaneously replicating lineages of SARS-CoV-2 is even less frequently reported despite being necessary for recombination (Bal et al., 2022; Rockett et al., 2022). SARS-CoV-2 inter-lineage recombination is only beginning to be investigated (Li et al., 2022), but has been demonstrated in the setting of COVID-19 (Francisco Junior et al., 2022; Lacek et al., 2022). There is evidence to suggest the emergence of the Omicron VOC can be traced to recombination between two ancestral SARS-CoV-2 lineages (Liu et al., 2022).

Thus, factors governing VOC emergence are multifactorial and influenced by a combination of genetic drift, recombination, host immunity, antiviral therapy, and duration of SARS-CoV-2 infection. In this work, we investigate SARS-CoV-2 coinfections among patients at a large referral center. Using a rapid genotyping method based on targeted RT-PCR and multiplex fragment analysis (Clark et al., 2021) we have detected two cases of Delta (B.1.617.2)/Omicron (B.1.1.529) BA.1 coinfections, and three cases of Omicron BA.1/BA.2 coinfection in hosts with various comorbidities. Identifying and characterizing coinfections with SARS-CoV-2 VOCs will reenforce evidence-based understanding of genomic change and infection patterns.

## Case: SARS-CoV-2 Coinfection with the Delta and Omicron BA.1 VOCs

**FIGURE 1:**
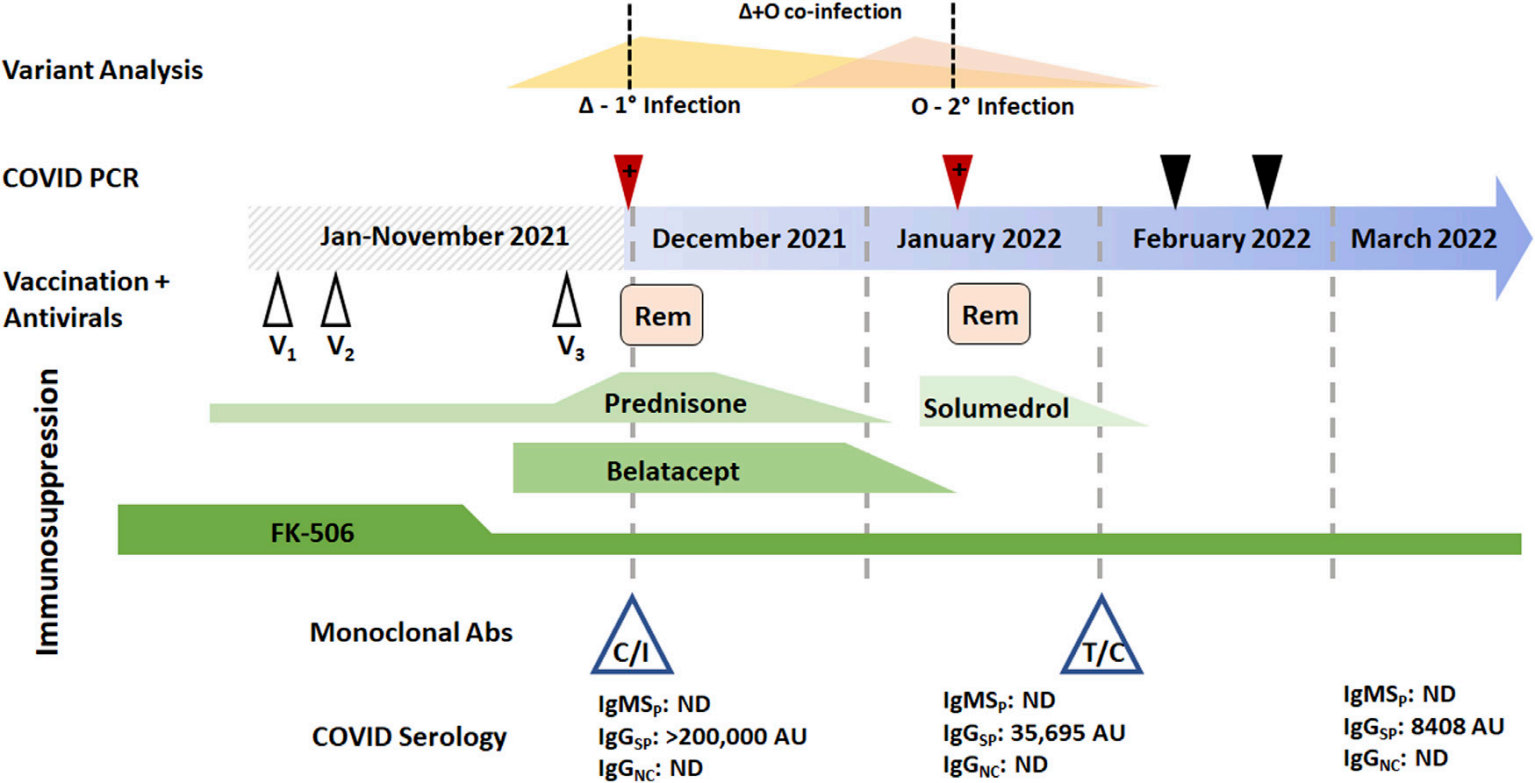
Longitudinal temporal analysis of the SARS-CoV-2 Delta/Omicron BA.1 coinfection reported in patient 1. Two positive RT-PCR samples (red arrowheads) and two negative RT-PCR samples were obtained between the end of November 2021 through February 2022 (black arrowheads). Prior vaccine (white arrowheads) and Remdesivir (pink boxes) are plotted when they were received in the disease course. Immunosuppressive regimens are represented (green boxes) with tapering as prescribed; FK-506 is tacrolimus. Monoclonal antibody therapy with either casirivimab/imdevimab (C/I) or tixagevimab/cilgavimab (T/C) is also denoted, as are serological values at the time of blood draw. Abbreviations: IgM_SP_, immunoglobulin M anti-spike; IgG_SP_, immunoglobulin G anti-spike; IgG_NC_, immunoglobulin G anti-nucleocapsid; 1^o^, primary infection; 2^o^, secondary infection.

A 76-year-old male who underwent bilateral lung transplantation 8 years prior due to idiopathic pulmonary fibrosis presented with fever and respiratory symptoms in November 2021 consistent with COVID-19. SARS-CoV-2 PCR was positive upon admission. Viral genotyping revealed mutations consistent with the Delta VOC which was epidemiologically dominant at the time of sampling. The patient’s immunosuppressive regimen included prednisone, tacrolimus, and monthly belatecept infusions. He had received three doses of the Pfizer/BioNTech COVID-19 vaccine prior to presentation. The patient was initially treated with Casirivimab/Imdevimab (Regeneron) followed by dexamethasone and remdesivir for 5 and 10 days, respectively (Figure 1). He was discharged without supplemental oxygen but reported continued exertional dyspnea and reduced forced expiratory volume (FEV1) from baseline (Figure 1).

Forty-five days later (early January 2022), he returned with fever, worsening shortness of breath, and productive cough. Respiratory RT-PCR was again positive for SARS-CoV-2, but given the proximity to prior infection, was initially thought to be a result of persistent viral shedding and alternative etiologies were sought. The patient was treated for hospital acquired pneumonia with piperacillin-tazobactam and vancomycin, and with methylprednisolone for acute cellular rejection. However, genotyping of the second PCR-positive specimen identified SARS-CoV-2 mutational signatures consistent with the presence of two unique VOCs. Delta (believed to be remaining from his initial infection) and Omicron BA.1 (secondary infection) were identified (Figure 3A). Following confirmation of active COVID-19, a course of Dexamethasone and Remdesivir was recommended. With discontinued antibiotics and tapered steroids, the patient was discharged without supplemental oxygen and a FEV1 near baseline.

### Laboratory findings

SARS-CoV-2 variant status is routinely determined for all RT-PCR-positive respiratory samples at our institution through a combination of whole genome sequencing (WGS) and genotyping by multiplex fragment analysis of RT-PCR products. Briefly, mutational hotspots of the SARS-CoV-2 genome are amplified with fluorescently labeled primers. These PCR amplicons are separated by capillary electrophoresis, and size differences determine the presence or absences of characteristic deletions. Nucleotide substitutions are determined though the competitive incorporation of primers with alternative florescent labeling (Clark et al., 2021).

**FIGURE 2:**
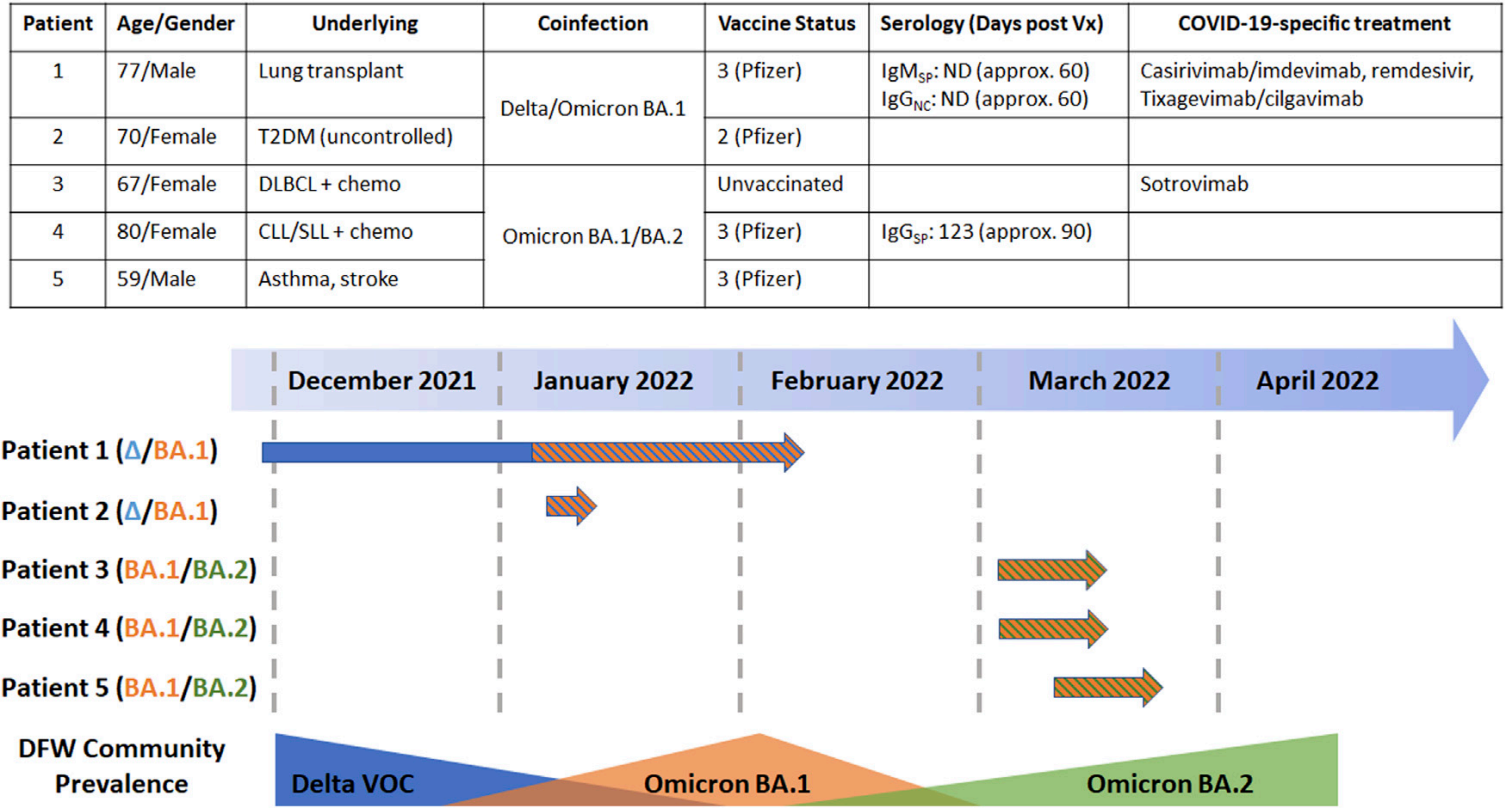
(Upper panel) Pertinent demographic and clinical characteristics of the five patients identified with SARS-CoV-2 coinfections in this work (Lower panel) Temporal analysis of the coinfections for each of the five patients identified in this work with respect to the community prevalence of VOCs as determined by prospective monitoring by multiplex fragment analysis. Abbreviations: T2DM, type-2 diabetes mellitus; DLBCL, diffuse large B-cell lymphoma; CLL/SLL, chronic lymphocytic leukemia/small cell lymphocytic leukemia; chemo, chemotherapy; Vx, vaccine; DFW, Dallas-Fort Worth.

This patient’s initial SARS-CoV-2 PCR-positive specimen contained only Delta VOC sequences, while the positive specimen from the subsequent admission contained mutational signatures consistent with a mixture of both Delta and the newly emerged Omicron BA.1 VOCs. At the time of both infections, the patient had undetectable levels of nucleocapsid-specific IgG of IgM antibodies, often see among patient groups receiving immunosuppressive therapies (Narasimhan et al., 2021; Thakkar et al., 2021; Vijenthira et al., 2021). As part of his management, the patient was given monoclonal antibody therapy (casirivimab and imdevimab for Delta), resulting in very high passive Spike IgG antibody levels which quickly diminished over the months following administration (Figure 1, Figure 2). Thus, he was later given prophylactic monoclonal antibodies (tixagevimab with cilgavimab, Figure 1).

**FIGURE 3:**
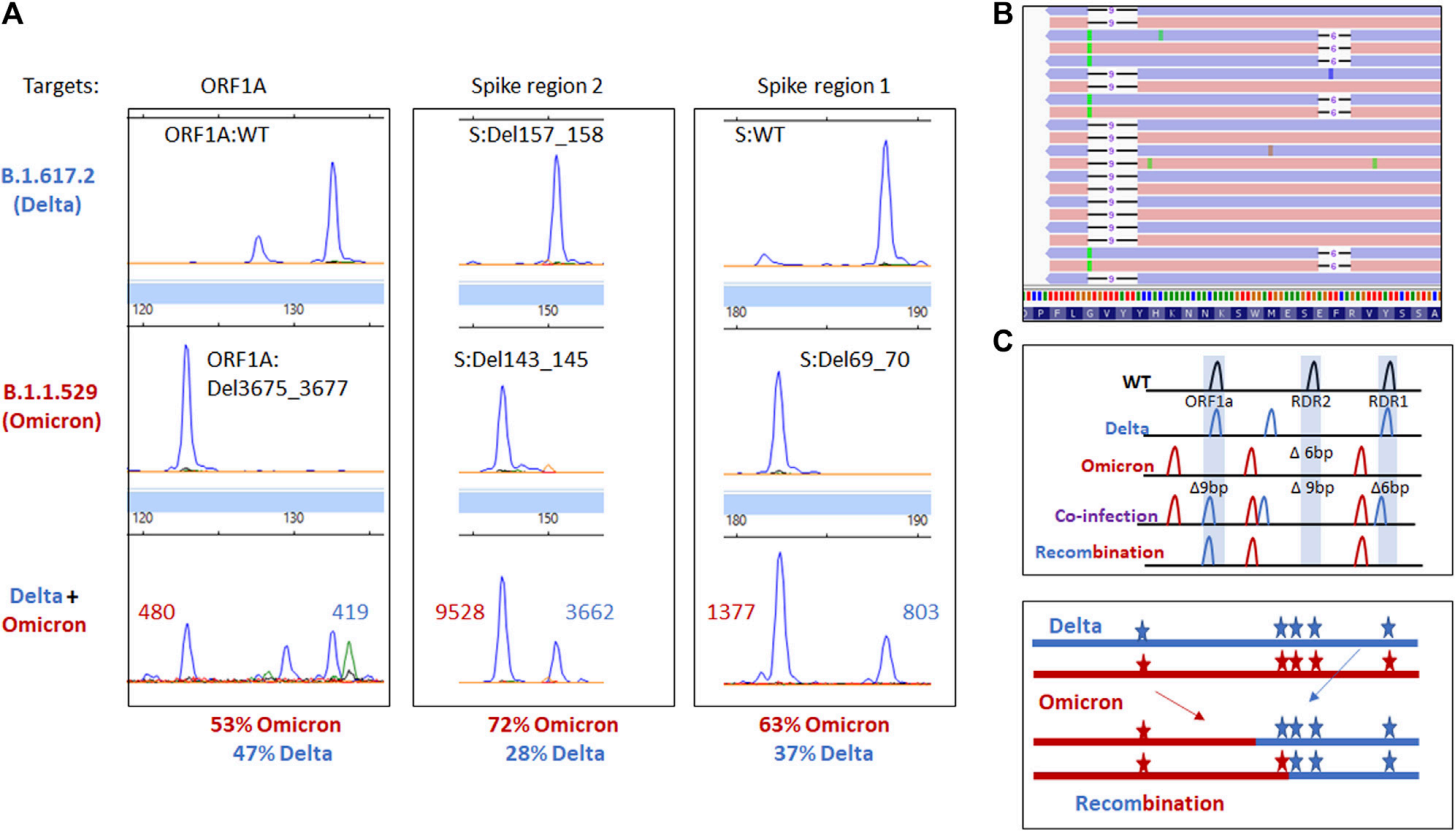
**(A)** Electrophoretogram of the ORF1A, Spike region 1, and Spike region 2 sites where different sized amplicons indicative of Delta (top) Omicron (middle) or coinfection (bottom) are present. **(B)** Next-generation sequencing reads were visualized in the Integrated Genome Viewer with read direction colored light red or violet. Integrated Genome Viewer (IGV) display shows deletions as indicated by a bar and single nucleotide variants have the variant nucleotide present. **(C)** Schematic illustrating the expected location of fragments for variants under normal, mixed, or recombined conditions.

Given the patient’s immunosuppressed and coinfected status, we wanted to determine if there was any evidence of recombination between the detected VOCs. WGS revealed mutations specific to Delta and Omicron BA.1 were found on independent reads using WGS. While a total of eight fragment analysis targets are used in genotyping, only three are highlighted here to demonstrate the difference in Omicron BA.1 and Delta VOCs. ORF1A detects the 3 amino acid deletion from ORF1A:Del3675_3677. Spike recurrently deleted region (RDR)1 and 2 detect S:Del69_70 and mutations in the 140–160 amino acid range, respectively. The Delta variant has a 2 amino acid deletion in Spike region 2 (S:Del157_158), while Omicron has a 3 amino acid deletion (S:Del143_145, Figures 3A,B). These data, paired with information from WGS, reveal no mutagenic signatures were identified to suggest recombination between the two lineages in this case (Figures 3B,C).

While we report the previous case in expanded detail, we also encountered an additional Delta/Omicron BA.1 coinfection in a 70-year-old female with uncontrolled type II diabetes (Figure 2). This case was notable as it occurred overlapped temporally with the coinfection at the beginning of January 2022 when both the Delta and Omicron BA.1 VOCs were present in the community in relative abundance. Upon presentation the patient was afebrile but complained of a loss of taste and persistent cough. The patient was previously vaccinated with two doses of the Pfizer/BioNTech COVID-19 vaccination almost a year prior, but her serological status was unknown. At the time of presentation, the patient was profoundly hyperglycemic which can impact immune function (Berbudi et al., 2020).

Type II diabetes is a risk factor for more severe COVID-19 (Hartmann-Boyce et al., 2021). Thus, while not having underlying malignancy or managed with immunosuppressive drugs as others in this case series, this patient’s immune status was likely dysregulated due to her comorbidity which can render her more susceptible to infection (Singh et al., 2022). This patient was managed without COVID-19 specific therapy and discharged 3 days later. As before, no mutational signatures consistent with SARS-CoV-2 recombination were identified in this patient’s respiratory specimens.

### Additional cases of SARS-CoV-2 coinfections

After identification of the two Delta/Omicron BA.1 coinfections, we prospectively monitored SARS-CoV-2 RT-PCR-positive specimens for additional instances of coinfections. The Omicron VOC emerged in November 2021 as the fifth VOC of the COVID-19 pandemic, which quickly replaced the Delta VOC as the predominant lineage in the United States. Three subvariants (named BA.1, BA.2, and BA.3) were identified at the relative onset of the outbreak, indicative of rapid evolutionary change (Desingu et al., 2022). While BA.1 was the predominant Omicron subvariant in the US at the beginning of the Omicron wave, BA.2 has since displaced it potentially due to enhanced transmissibility, growth kinetics, and capacity to subvert vaccine-associated immunity (Callaway, 2022). Given these two variants were present in the population simultaneously (like the Delta and Omicron BA.1 VOCs previously), we sought to identify Omicron BA.1/BA.2 coinfections among our local population (Figure 2). Figure 2 relates the clinical and temporal characteristics of these co-infections, highlighting how they occur at times where there is an overlap in variants, which we hypothesize as a plausibility for the occurrence of co-infections.

**FIGURE 4:**
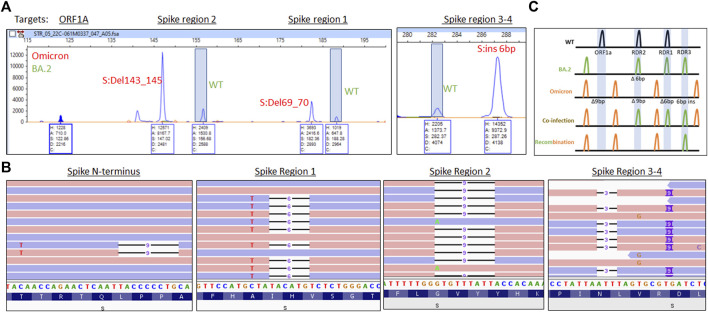
**(A)** Electrophoretogram of ORF1A, Spike region 1, Spike region 2, and Spike region three to four sites where different sized amplicons indicative of Omicron (red) and BA.2 (green) coinfection are present. **(B)** Next-generation sequencing reads were visualized in the Integrated Genome Viewer with read direction colored light red or violet. Deletions are indicated by a bar, single nucleotide variants have the variant nucleotide present, and insertions are highlighted by a purple box. **(C)** Schematic illustrating the expected location of fragments for variants under normal, mixed, or recombined conditions.

Respiratory samples from three unique patients revealed multiple peaks amplified for RDR1 (WT and 6 base pair deletion), RDR2 (WT and 9 base pair deletion), RDR3-4 (WT and a 6 base pair insertion, Figure 4). This fragment pattern could only occur when Omicron BA.1 and BA.2 genomic RNAs were present in the same reaction from a coinfection. To rule out the possibility of contamination, RNA was re-extracted from each sample and analysis was repeated yielding identical results. The co-occurrence of amplicons specific to the Omicron BA.1 and BA.2 subvariants indicates a coinfection, rather than recombination, has occurred in these samples. WGS determination of a coinfection requires mutations be within 100–150 base pairs of each other to observe co-occurrence of mutations on the same read strand based on the length of the amplicons in the sequencing kit used. Here, we focused on the spike gene where multiple differences in mutations were found.

WGS revealed separate forward and reverse reads (colored red and blue respectively, Figure 4B) for the Omicron BA.1 mutations (S:69_70Del and A67V) and BA.2 (no mutations present) in the RDR1 region. In the RDR2 region, the Omicron deletion (S:143_145Del) is present with alternative reads from the BA.2 variant (S:G142D). In the RDR3-4 region, separate reads are observed with mutations indicative of for Omicron (S:211Del, 214 insEPE) and BA.2 (S:V213G). Finally, in the N-terminal portion of the S gene, an S:L24S, S:25_27Del variants from BA.2 are present with sequence consistent with the Omicron variant (no mutation). These findings are consistent with Omicron BA.1/BA.2 coinfection without detectable recombination in these patients (Figure 4C).

Clinically, these BA.1/BA.2 co-infections were characterized by ages from 59 to 80 years old (Figure 2, Upper panel). Two patients were vaccinated, two had hematologic malignancies, and the other case only had a history of asthma. The unvaccinated person with diffuse large B cell lymphoma on chemotherapy was additionally given sotrovimab monoclonal antibody therapy to prevent worsening symptoms. All patients had a mild course and overcame their symptoms within 2 weeks.

## Discussion

Here we describe five cases of SARS-CoV-2 coinfection with unique VOCs: two cases of Delta/Omicron BA.1 coinfection and three cases of Omicron BA.1/BA.2 coinfection. While infectivity and replication dynamics of individual SARS-CoV-2 genetic lineages continue to be elucidated, significantly less is known concerning coinfections with unique SARS-CoV-2 variants. The paucity of information in the literature is likely multifactorial, given an unpredictable epidemiological pattern of variant emergence and nonuniform application or access to sequencing or genotyping methods. These three early cases of Omicron BA.1/BA.2 coinfections temporally coincide with the Delta/Omicron BA.1 coinfection. It is reasonable to predict such coinfections will continue to occur due to overlapping emergence of SARS-CoV-2 VOCs, or when multiple lineages are present at a given time within in the community.

Prior work has identified coinfection as an impetus for recombination between different SARS-CoV-2 variants (Jackson et al., 2021; He et al., 2022). Indeed, IC hosts with impaired ability to clear SARS-CoV-2 infections may increase the chances of coinfection/recombination. The serological responses among IC patients are dampened as exemplified by the lack of Nucleocapsid IgG antibody response after months of primary and secondary infection in this lung transplant patient. The high anti-Spike IgG levels were due to monoclonal antibody use. Viral adaptation in this setting is also a concern as these hosts are often recipients of anti-SARS-Cov-2 therapy and expanded of SARS-CoV-2 genomic diversity has been reported in this setting (Chen et al., 2021). Interrogations of the underlying mechanisms contributing to this diversity are only beginning to be undertaken.

Host metabolic proteins Sirtuin 1 and 5 (SIRT1 and SIRT5) have been recently shown to interact with SARS-CoV-2 Nsp14 and generating a lethal phenotype of SARS-CoV-2 with effective replication and/or long-term propagation (Walter et al., 2022). Importantly, the expression and activity of SIRT1 was shown to be enhanced by the steroid, prednisolone (Sung et al., 2020), which the case patient has been receiving since transplant at a dose of 7.5 mg daily until the November COVID diagnosis and continued with 60 mg daily following this initial diagnosis. Thus, any such alterations in host cell pathways due to IC and/or immunosuppressive (IS) drugs that can interfere with and dampen the SARS-CoV-2 nsp14-ExoN is likely to hamper the intrinsic fidelity and contribute to high-level mutagenesis.

The small number of cases observed prevents generalizable conclusions concerning coinfection protracting disease course or severity. However, a few overarching commonalities can be identified between the patients in these cases. Protracted disease presents an opportunity for additional exposure or the emergence of new variants. Of note, it has been reported that persistently infected IC patients accumulate amino acid substitutions or deletions in different regions of human SARS-CoV-2 spike protein and thus are conceived to be a source of new immune-escape viral variants (Avanzato et al., 2020; Choi et al., 2020; Weigang et al., 2021). Also, CD4^+^ T-cell depletion (<20 CD4^+^ T-cell counts) as found in IC persons, including persons living with HIV/AIDS, has been determined to increase the risk of giving rise to SARS-CoV-2 resistance mutations (Hoffman et al., 2021; Cele et al., 2022). In contrast, an absent immune response does not reject virus and thus there is no competitive advantage for acquiring resistance mutations. Furthermore, we have learned that these cases of SARS-CoV-2 coinfection are most likely to occur in times when there is a diversity of variants in circulation. Thus, it is likely that coinfections will continue to occur and methods to monitor this should be considered.

The most used variant detection method is currently WGS. However, criteria for calling variants are that mutations must be detected at >50% or higher levels of variant allele frequencies. Additionally, due to biases introduced in amplicon-based library preparation, whole genome sequencing cannot accurately quantify differences in variant levels. This cut-off prevents minor allele-frequencies to be counted for detection of coinfections. Genotyping methods offer a sensitive alternative where multiple mutations can be detected at once provided a wild-type and mutant allele can be detected simultaneously. In this scenario, fragment analysis is superior, because it can detect multiple types of deletions simultaneously. This method of multiplex SARS-CoV-2 genotyping by fragment analysis lends itself to not only detecting concurrent variant mutational signatures but also allows relative quantitation of the genomic material present. This quantitation can be cross applied to the CT value to infer whether an infection is active or not. For our patient, a 25% decrease in Delta nucleic acid corresponds with a 4-fold difference (2 CT value difference), which with a CT value of 27 places the prevalence of both variants in the range of active infection and thus, less likely to be due to the presence of shedding dead virus.

The principal limitation to widespread adoption of this approach is the upfront cost of some capillary electrophoresis devices. However, newer, smaller, bench-top options that are attractive and less expensive can overcome this challenge.

Importantly, SARS-CoV-2 genomic recombination can be rapidly and economically screened by fragment analysis, alleviating the need for the bioinformatic expertise or financial burden associated with traditional NGS pipelines. The variant identification program, Pangolin, can be programed to distinguish recombinant lineages including the recently described XD, XE, and XF variants. Nextclade, and non-amplicon-based platforms and bioinformatic tools such as RAT (Recombination Analysis Tool) or RDP (Recombination Detection Program) may also aid in the characterization of SARS-CoV-2 recombinatory events. However, by placing targets across the genome, this fragment analysis assay may detect recombination. Most assays just target the spike gene and recombination can occur across the entire SARS-CoV-2 genome. We conclude that SARS-CoV-2 coinfections will continue and represent a source of viral evolution, which in many cases can be effectively monitored using a fragment analysis genotyping approach.

## Data Availability

The datasets for this article are not publicly available due to concerns regarding participant/patient anonymity. Requests to access the datasets should be directed to the corresponding author.
